# Highly Porous Composite Scaffolds Endowed with Antibacterial Activity for Multifunctional Grafts in Bone Repair

**DOI:** 10.3390/polym13244378

**Published:** 2021-12-14

**Authors:** Ana S. Neto, Patrícia Pereira, Ana C. Fonseca, Carla Dias, Mariana C. Almeida, Inês Barros, Catarina O. Miranda, Luís P. de Almeida, Paula V. Morais, Jorge F. J. Coelho, José M. F. Ferreira

**Affiliations:** 1 Department of Materials and Ceramic Engineering/CICECO—Aveiro Institute of Materials, University of Aveiro, 3810-193 Aveiro, Portugal; sofia.neto@ua.pt; 2 Department of Chemical Engineering, CEMMPRE, University of Coimbra, 3030-790 Coimbra, Portugal; ppereira@ipn.pt (P.P.); jcoelho@eq.uc.pt (J.F.J.C.); 3 IPN, Instituto Pedro Nunes, Associação para a Inovação e Desenvolvimento em Ciência Tecnologia, Rua Pedro Nunes, 3030-199 Coimbra, Portugal; 4 Department of Life Sciences, CEMMPRE, University of Coimbra, 3001-401 Coimbra, Portugal; carla_spd@hotmail.com (C.D.); mcalmeida@uc.pt (M.C.A.); pvmorais@ci.uc.pt (P.V.M.); 5 CNC—Center for Neuroscience and Cell Biology, University of Coimbra, 3004-504 Coimbra, Portugal; ines.barros2095@gmail.com (I.B.); csmiranda@cnc.uc.pt (C.O.M.); luispa@cnc.uc.pt (L.P.d.A.); 6 CIBB—Center for Innovative Biomedicine and Biotechnology, University of Coimbra, 3004-504 Coimbra, Portugal; 7 IIIUC—Institute for Interdisciplinary Research, University of Coimbra, 3030-789 Coimbra, Portugal; 8 Faculty of Pharmacy, University of Coimbra, 3000-548 Coimbra, Portugal; 9 Viravector—Viral Vector for Gene Transfer Core Facility, University of Coimbra, 3004-504 Coimbra, Portugal

**Keywords:** cuttlefish bone, biphasic calcium phosphate, polymeric coatings, rifampicin, drug delivery system

## Abstract

The present study deals with the development of multifunctional biphasic calcium phosphate (BCP) scaffolds coated with biopolymers—poly(ε-caprolactone) (PCL) or poly(ester urea) (PEU)—loaded with an antibiotic drug, Rifampicin (RFP). The amounts of RFP incorporated into the PCL and PEU-coated scaffolds were 0.55 ± 0.04 and 0.45 ± 0.02 wt%, respectively. The in vitro drug release profiles in phosphate buffered saline over 6 days were characterized by a burst release within the first 8h, followed by a sustained release. The Korsmeyer–Peppas model showed that RFP release was controlled by polymer-specific non-Fickian diffusion. A faster burst release (67.33 ± 1.48%) was observed for the PCL-coated samples, in comparison to that measured (47.23 ± 0.31%) for the PEU-coated samples. The growth inhibitory activity against *Escherichia coli* and *Staphylococcus aureus* was evaluated. Although the RFP-loaded scaffolds were effective in reducing bacterial growth for both strains, their effectiveness depends on the particular bacterial strain, as well as on the type of polymer coating, since it rules the drug release behavior. The low antibacterial activity demonstrated by the BCP-PEU-RFP scaffold against *E. coli* could be a consequence of the lower amount of RFP that is released from this scaffold, when compared with BCP-PCL-RFP. In vitro studies showed excellent cytocompatibility, adherence, and proliferation of human mesenchymal stem cells on the BCP-PEU-RFP scaffold surface. The fabricated highly porous scaffolds that could act as an antibiotic delivery system have great potential for applications in bone regeneration and tissue engineering, while preventing bacterial infections.

## 1. Introduction

Bacterial infections are one of the main problems associated with the implantation of conventional medical devices and can lead to increases in patient morbidity and mortality [1]. Bacterial infections are equally seen in tissue engineering approaches using scaffolds [2]. Irreversible adhesion of microorganisms creates a biofilm that protects bacteria from phagocytosis and antibiotics [3,4]. Thus, inhibiting irreversible bacterial adhesion is one of the most important prior steps for granting a successful implantation procedure. Bacterial infections could be prevented using scaffolds that allow early local antibiotic administration. The effectiveness of a delivery system is highly dependent on the control of the drug release profile. Since there is a high risk of infection immediately after implantation, the delivery system should promote an initial burst release of the antibiotic. This initial burst release should be followed by a sustained release to avoid a latent infection [3].

Calcium phosphates (CaP) are the most common biomaterials used in bone tissue engineering due to their similarity to the mineral component of bone and their excellent bioactivity. Nowadays, biphasic calcium phosphate (BCP) represents the gold standard of CaP biomaterials [5]. BCP usually combine relatively stable hydroxyapatite (HA) with a more soluble β-tricalcium phosphate (β-TCP) phase, thus allowing better control over the bioactivity and biodegradability of the scaffold. In this way, the stability of the material is ensured during the ingrowth of the bone [6]. Despite all the potential of CaP materials, they have some disadvantages, mainly their brittleness and low strength. These disadvantages can be mitigated by applying polymeric coatings to improve the robustness of the inorganic material [7]. In this context, synthetic polymers offer a number of advantageous over the natural ones, including the ability to easily adjust their physicochemical properties, and more reproducible synthesis and production processes [8]. The synergistic combinations between CaP and polymers have also been explored for drug delivery in bone tissue engineering [9,10,11]. Despite the ability of CaP scaffolds to incorporate pharmaceutical agents by surface adsorption, [12,13] they have low efficiency as sustained release systems. In addition, the relatively high temperatures required to obtain high skeletal density and suitable mechanical properties tend to reduce the surface area available for adsorption, making them potentially unsuitable for drug incorporation and release [14]. These drawbacks can be overcome by incorporating the desired active pharmaceutical ingredient into a polymeric coating [14]. Drug release from a polymeric coating is characterized by an initial burst release followed by a sustained release [15]. Several factors influence the drug release profile, namely, coating degradation, interaction with the polymer, and diffusion of the drug. Moreover, the properties of the scaffolds such as porosity, pore size and interconnectivity play a crucial role in the drug release profile [14]. 

**Figure 1:**
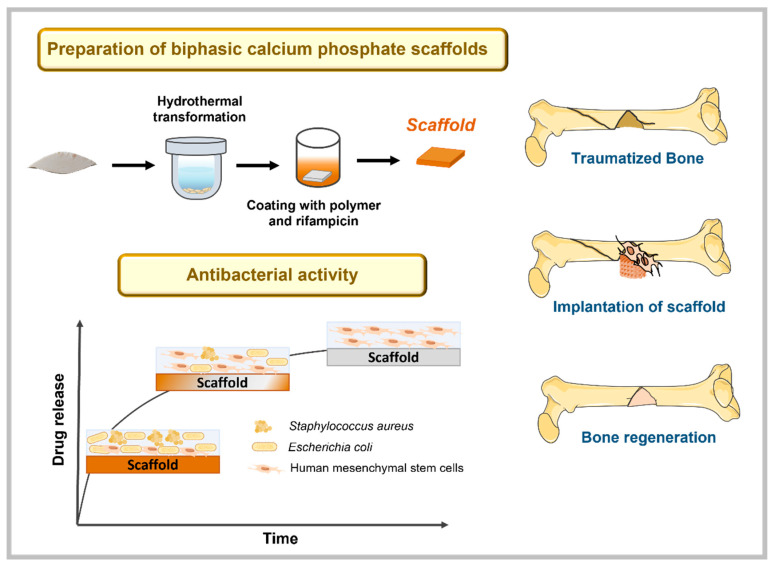
Overall strategy for the preparation of multifunctional biphasic calcium phosphate scaffolds coated with polymeric materials and with drug delivery properties.

Cuttlefish bone (CB) has a unique architecture with about 93% porosity [16]. The successful hydrothermal conversion (HT) of CB into BCP scaffolds has been reported previously [17]. Due to the brittleness and low strength of the hydrothermally converted HA scaffolds, polymeric reinforcement coatings have been reported elsewhere [18,19,20]. In our previous work, poly(ε-caprolactone) (PCL) or poly(ester urea) (PEU) coatings were used to improve the mechanical properties [17]. PCL is one of the most used synthetic polymers in bone tissue engineering. It is a biocompatible polymer with good mechanical properties and its degradation products are non-toxic [21,22]. On the other hand, PEU is a synthetic polymer with promising properties. The presence of α-amino acids improves cell-material interactions and enables a further functionalization that represents a powerful tool in the biomedical field [23,24]. In the present work, a further step was taken and the polymer-coated BCP scaffolds were investigated as a vehicle for the uptake and release of an antibiotic to avoid bacterial infection, thus obtaining multifunctional scaffolds. With this purpose, BCP scaffolds derived from CB were synthesized, heat treated and coated with PCL or PEU solutions, in which the antibiotic rifampicin (RFP), was dissolved (Figure 1). RFP has a broad-spectrum against Gram-positive and-negative bacterial strains. Moreover, it binds to the enzyme RNA-polymerase and blocks the bacterial DNA function [25]. As one of the most potent and broad-spectrum antibiotics, RFP has been explored as a pharmaceutical agent to prevent the formation of biofilm [26,27,28].

## 2. Materials and Methods

### 2.1. Preparation of BCP Scaffolds

The bones of cuttlefish, *Sepia officinalis*, were cut into cylinders of approximately 6 mm in diameter and 3 mm in height. Using differential and gravimetric thermal analysis (DTA/TG, Labsys Setaram TG-DTA/DSC, Caluire-, France, heating rate of 10 °C min^−1^), the exact amount of CaCO_3_ in the CB (calcium precursor) was calculated. The CB cylinders were then subjected to hydrothermal transformation (HT) in the presence of a phosphorous precursor. Briefly, the samples were sealed in a stainless-steel autoclave lined with poly(tetrafluorethylene) (PTFE) with the required volume of an aqueous solution of (NH_4_)_2_HPO_4_ (Panreac AppliChem, Castellar del Vallès, Spain) for 24 h at 200 °C. The obtained scaffolds were then subjected to heat treatment to remove the organic matter at 700 °C for 1 h at a heating rate of 0.5 °C min^−1^, followed by a heating ramp of 2 °C min^−1^ up to 1200 °C and a dwelling time of 2 h at this temperature for sintering.

### 2.2. Preparation of Polymeric Coated Scaffolds Loaded with RFP

The sintered BCP scaffolds were coated with PCL (Perstorp Specialty Chemicals AB, Perstorp, Sweden, CAPA^TM^ 6800 M_n_ = 80,000 g mol^−1^) and PEU (Mn = 63,000 g mol^−1^). The polymer solutions were prepared at a concentration of 5% (*w*/*v*); PCL was dissolved in dichloromethane (Sigma, Darmstadt, Germany), while PEU was dissolved in chloroform (Fisher Scientific, Loughborough, UK). To improve the solubility of the PEU, 2% (*v*/*v*) of *N*,*N*′-dimethylformamide (Sigma-Aldrich, Darmstadt, Germany) was added to the chloroform solution. The scaffolds were coated by the dip coating method using a vacuum system for 20 min at the pressure of 0.4 bar. Two different samples were obtained: BCP coated with PCL (BCP-PCL) and BCP coated with PEU (BCP-PEU). To obtain RFP loaded samples, RFP powder (Panreac AppliChem, Castellar del Vallès, Spain) was dissolved in the polymer solution at a concentration of 1.5 mg.mL^−1^. The scaffolds coated with RFP-containing PCL or PEU solutions, BCP-PCL-RFP or BCP-PEU-RFP, respectively, were obtained following the same procedure described above for preparing the BCP-PCL and the BCP-PEU samples. The incorporated RFP content was determined by immersing the scaffolds in dimethyl sulfoxide (DMSO), which allowed for the complete dissolution of the drug. Subsequently, the RFP content was measured by UV-Vis spectrophotometry at the wavelength of 338 nm. A calibration curve (Figure S1) was plotted within an appropriate RFP concentration range in DMSO.

### 2.3. Characterization of the Obtained Scaffolds

All of the scaffold samples (BCP, BCP-PCL, BCP-PEU, BCP-PCL-RFP and BCP-PEU-RFP) were characterized by Fourier transform infrared spectroscopy (FTIR), X-ray diffraction (XRD) and differential scanning calorimetry (DSC). FTIR spectra were acquired at room temperature (RT) using an Agilent Technologies Carey 630 spectrometer (Agilent Technologies, Inc., Santa Clara, CA, USA) equipped with a Golden Gate Single Reflection Diamond ATR. Data were recorded in a range from 650 to 4000 cm^−1^ with a spectral resolution of 4 cm^−1^ and 64 accumulations. XRD measurements were performed in a high-resolution X-ray diffractometer (PANalytical X’Pert Pro, Malvern Panalytical, Los Altos, CA, USA) with Cu Kα radiation (λ = 1.5406 Å) using a step-scanning mode with a 2θ angle from 10° to 100° and a step size of 0.0260° per second. DSC measurements were performed in a Netzsch DSC-214 (Netzsch, Selb, Germany) under a nitrogen atmosphere using an aluminium pan containing approximately 5 mg of sample. A heating rate of 10 °C min^−1^ was used within a temperature range from −40 °C to 500 °C.

### 2.4. In Vitro RFP Release Study

In order to evaluate the in vitro RFP release profile, the scaffolds were placed in glass tubes containing 2 mL of PBS and incubated at 37 °C in a shaker for different time points (10 and 30 min; 1, 2, 6, 8, and 24 h; 2, 3, 4, 5 and 6 days). The amount of RFP released into the medium was measured by UV-Vis spectrophotometry at wavelength 338 nm (Figure S2). Measurements were performed in triplicate. The kinetic release of RFP from the polymer coated BCP scaffold was modelled using the Korsmeyer–Peppas model (Equation (1))

(1) $$\frac{M_{t}}{M_{\infty }}=kt^{n}\;\left(\frac{M_{t}}{M_{\infty }}\;\leq 0.6\right)$$

where *M_t_* and *M*_∞_ are the amounts of drug released at time *t* and infinity, respectively; *k* is the release constant and *n* is the release exponent and is related to the release mechanism [29].

### 2.5. Antibacterial Activity Assay

Antibacterial assays of the RFP-loaded and unloaded scaffolds were performed against Gram-negative, *Escherichia coli* ATCC25922, and Gram-positive, *Staphylococcus aureus* ATCC25923. The bacterial strains were grown at 37 °C for 24 h in Luria-Bertani (LB) medium. A bacterial suspension was then prepared in 10 mL of PBS solution with turbidity adjusted to 0.5 according to the McFarland standard (1.5 × 10^8^ CFU mL^–1^). The obtained suspension was diluted 10-fold and then 375 µL was mixed with 1125 µL of LB medium and added to the wells containing the different polymer (PCL or PEU) coated BCP scaffolds with or without RFP. The well without any scaffold was used as a positive control. The samples were incubated with an orbital shaker at 115 rpm and 37 °C. The number of viable cells remaining in the culture medium after 24, 48 and 72 h was analysed by the spread plate method. Briefly, 100 µL of bacterial suspension was taken from the wells and spread evenly on LB agar plates. At 72 h incubation, the scaffolds in contact with the bacterial suspension, were gently washed twice with PBS and the cells attached were detached by sonication for 10 min. The obtained bacterial suspension was also spread on LB agar plates. Finally, the LB dishes were incubated overnight at 37 ºC and the number of colony-forming units (CFUs) was determined. The experiments were repeated in triplicate and with two replicates in each experiment.

### 2.6. Isolation and Culture of Human Mesenchymal Stem Cells from Umbilical Cord Matrix

Human umbilical cords from healthy donors after birth, with parental consent, were kindly donated by Crioestaminal Saúde e Tecnologia (Biocant Park, Cantanhede, Portugal). The umbilical cords were stored in sterile 50 mL tubes at RT between 12 and 48 h before tissue processing. The samples were cut into small pieces of approximately 5 cm. The pieces obtained were washed with sterile PBS to remove blood. The umbilical veins were also washed to remove blood and blood clots. To avoid contamination by endothelial cells, the umbilical veins and arteries were also removed. Samples were then dried in tissue culture plates to promote adhesion of the fragment to the polystyrene surface, and, after adhesion, human mesenchymal stem cells (hMSCs) proliferation medium (alpha-MEM without ribonucleosides and deoxyribonucleosides (GIBCO™ Invitrogen Corporation, Carlsbad, CA, USA) supplemented with 10% foetal bovine serum (Cytiva HyClone™ Fetal Bovine Serum (FBS) U.S. Origin, Fisher Scientific, Loughborough, UK), 1% penicillin/streptomycin and 1% amphotericin B (GIBCO™ Invitrogen Corporation, Carlsbad, CA, USA)) was added to the cell culture plate. Samples were cultured at 37 °C with 5% CO_2_ and 95% humidity, for 10 days until hMSCs migrated from the umbilical cord matrix and defined colonies formed. Finally, fragments were removed from the umbilical matrix and cells were passaged. Passages 2–4 were used in all further experiments.

### 2.7. In Vitro Cytocompatibility Assays

Before seeding the cells, all scaffolds were sterilized by UV-irradiation on both sides for 15 min and pre-wetted in a culture medium for 6 h. The culture medium was removed and hMSCs were seeded at a density of 1 × 10^5^ per scaffold onto the top of the scaffolds. To promote cell adhesion, the seeded scaffolds were incubated for 2 h followed by the addition of 1 mL of culture medium. The culture medium was changed every 2–3 days. Cells seeded into the scaffolds were cultured in an incubator at 37 °C and a humidified atmosphere (5% CO_2_ and 95% air) for a period of 1–14 days.

### 2.8. Cell Viability and Proliferation

Cell viability and proliferation of seeded scaffolds with and without RFP were determined using the CellTiter 96^®^ AQueous One Solution Cell Proliferation colorimetric assay (MTS assay, Promega Corporation, Madison, WI, USA) according to the manufacturer’s instructions. After 1, 7 and 14 days of incubation, the culture medium was removed and 500 μL of serum-free culture medium containing MTS reagent (10%) was added to each well and incubated for 3 h at 37 °C and 5% CO_2_. Then, 100 µL of each well (in quadruplicate) were transferred to a 96-well plate and the absorbance was measured at 490 nm. A negative control (untreated cells), i.e., cells cultured without being exposed to the scaffolds, was performed. Cell viability was calculated as the percentage of viable cells relative to the untreated control cells, which were considered to have 100% viability.

### 2.9. Cell Attachment

To evaluate cell adhesion and morphology of attached cells to scaffolds with and without RFP, SEM was used. After 14 days of culturing, the seeded scaffolds were rinsed with PBS and fixed with paraformaldehyde (4% in PBS 1×) for 1 h at RT. After fixation, samples were washed again with PBS 1× and were dehydrated stepwise through ethanol solutions (30, 50, 70 and 90%) for 15 min with a final dehydration in absolute ethanol for 30 min. The dried cell-seeded scaffolds were sputter-coated with a gold layer before visualization by SEM. SEM images were acquired at various magnifications, at accelerating voltage of 10 kV, using a high-resolution field emission scanning electron microscope, with EDS, WDS (STEM ZEISS, Merlin, Oberkochen, Germany).

### 2.10. Statistical Analysis

Each experiment was performed in triplicate, using two replicates in each experiment. Results are expressed as mean ± standard deviation (SD) and two-way-analysis of variance (ANOVA) was used for statistical analysis. Results were considered statistically different when the *p*-value was less than 0.001. Data analysis and statistical tests were performed in GraphPad Prism 6.03 software (San Diego, CA, USA).

## 3. Results and Discussion

### 3.1. Characterization of the Scaffolds

**Figure 2:**
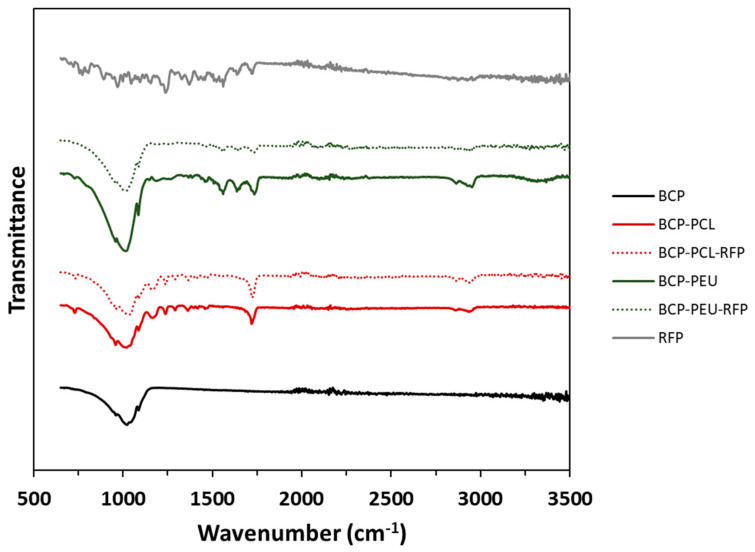
FTIR-ATR of BCP scaffolds, uncoated and after coating with different polymers (BCP-PCL and BCP-PEU) and further loaded with RFP (BCP-PCL-RFP and BCP-PEU-RFP).

The chemical groups present in the BCP scaffolds uncoated, and coated with PCL (BCP-PCL), PEU (BCP-PEU), and further RFP loading (BCP-PCL-RFP, BCP-PEU-RFP) were investigated by FTIR-ATR (Figure 2).

**Figure 3:**
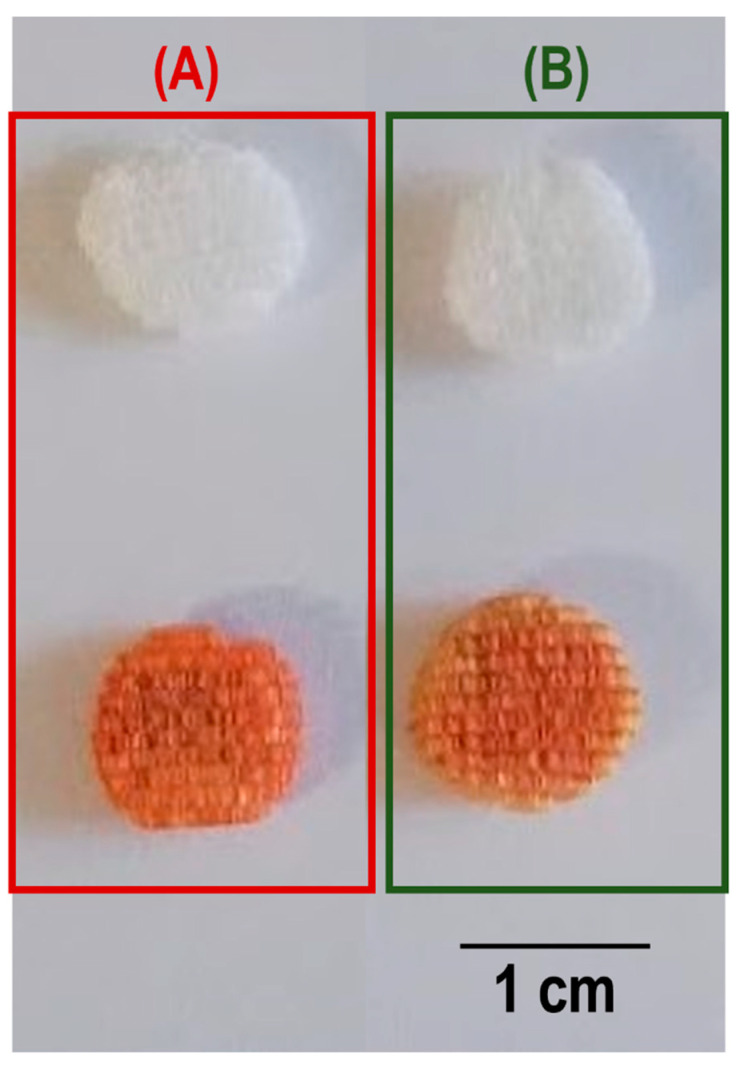
BCP scaffolds coated with different polymers (PCL (**A**) and PEU (**B**)—white), and further loaded with RFP (burnt orange).

The BCP scaffolds exhibit the characteristic bands of –PO_4_ groups. The band at 960 cm^−1^ is related to the ν_1_–PO_4_ stretching, and the stretching ν_3_–PO_4_ mode shows intense bands at 1028 and 1082 cm^–1^. The characteristic –OH band at 3575 cm^−1^ is not observed in the FTIR-ATR spectrum. This could be a consequence of the partial de-hydroxylation of HA at 1200 ºC and its absence in the β-TCP phase. The polymer coated scaffolds show an overlap of the BCP spectrum with the characteristic spectra of the polymers. In the PCL coated scaffolds, the characteristic bands of C=O stretching vibrations (ester linkage) can be observed at 1720 cm^−1^, CH_2_ stretching modes at 2946 and 2896 cm^−1^ and bending modes at 1362, 1399 and 1457 cm^−1^. At 1239, 1041 and 1107 cm^−1^ the bands associated with C–O–C stretching vibrations are observed. The C–O and C–C stretching of the amorphous and crystalline phases appear at 1166 and 1293 cm^–1^, respectively. In turn, the PEU-coated samples also exhibit the characteristic PEU bands. The band at 1560 cm^−1^ is associated with the bending of the N–H group and the stretching of the C–N group of the urea bond. The band at 3370 cm^−1^ corresponds to the stretching vibration of the N–H group of the urea linkage. The C=O vibration of ester and urea is at 1735 and 1636 cm^−1^, respectively. RFP has its characteristic bands of C=O at 1559 cm^−1^, furanone (C=O) at 1638 cm^−1^, acetyl (C=O) at 1720 cm^−1^ and amide (N–CH_3_) at 2890 cm^−1^ [30]. The addition of RFP did not change the spectra of the samples. In fact, the spectra before and after incorporation of RFP were identical. Although the presence of RFP cannot be detected by FTIR, it can be clearly seen in Figure 3 where the samples with RFP change colour from white to burnt orange.

### 3.2. In Vitro RFP Release Study

**Figure 4:**
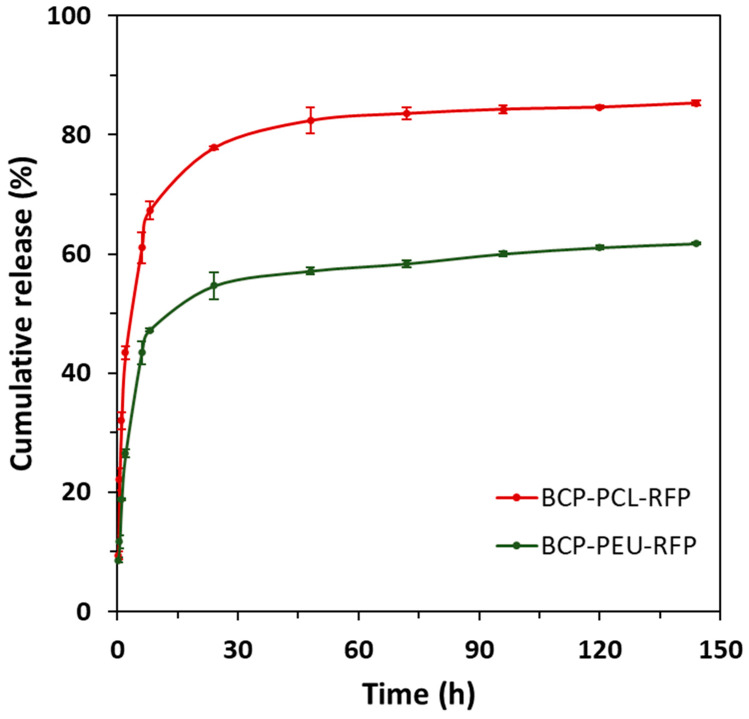
In vitro release profiles of RFP from the BCP scaffolds coated with PCL and PEU, in PBS (pH = 7.4), at 37 °C.

The RFP contents determined for PCL and PEU-coated samples were 0.55 ± 0.04% and 0.45 ± 0.02%, respectively. The loading capacity of the PCL coated scaffolds was significantly higher compared to that of PEU. The drug release profiles were determined over a period of 6 days and the results are shown in Figure 4. The cumulative release profiles for the different compositions are roughly similar, being characterized by two stages, an initial burst release (~8 h) followed by a sustained release period. Despite this similarity, the fraction of RFP released from the scaffolds revealed to be dependent on the polymer coating used. Indeed, the RFP release from BCP-PCL-RFP (67.33 ± 1.48%) was significantly higher during the first 8 h compared to the BCP-PEU-RFP systems, which released only 47.23 ± 0.31%. After 6 days, the total RFP content released reached 85.33 ± 0.36% and 61.73 ± 0.16% for BCP-PCL-RFP and BCP-PEU-RFP, respectively. The RFP release data revealed a good agreement with the Korsmeyer–Peppas model described in Equation (1), as deduced from the fitting parameters reported in Table 1. The values of release exponent (*n*) ranging from 0.46 to 1, indicate a non-Fickian diffusion for cylindrical samples. Moreover, the calculated values of constant (*k*) were 0.3058 and 0.1841 for BCP-PCL-RFP and BCP-PEU-RFP, respectively. The correlation coefficient was kept above 0.93 for all the scaffolds compositions.

### 3.3. Antibacterial Activity Assays

The antibacterial activity data was assessed by determining the time-dependent reduction of cells of *E. coli* and *S. aureus* in the presence of the scaffolds, using the CFU determination by the spread plate method (Table 2). The polymer coated samples, without RFP, did not exhibit any antimicrobial activity. Regarding the polymer coated scaffolds, loaded with RFP, all were effective in reducing the growth of *S. aureus*. Nevertheless, with *E. coli* the BCP-PEU-RFP scaffold did not show the ability to inhibit bacterial growth, contrarily to what was observed with BCP-PEU-RFP. After 24 h of incubation with *E. coli*, the BCP-PCL-RFP samples showed higher reduction in bacterial growth compared to BCP-PEU-RFP samples. After 72 h of incubation, the BCP-PCL-RFP resulted in a 100% reduction in the number of *E. coli*. For the BCP-PCL-RPF few attached cells (both *E.coli* and *S. aureus*) were observed after the 72 h, evidenced the difficulty of both strains in colonize these scaffolds.

### 3.4. Viability and Proliferation of Human Mesenchymal Stem Cells

**Figure 5:**
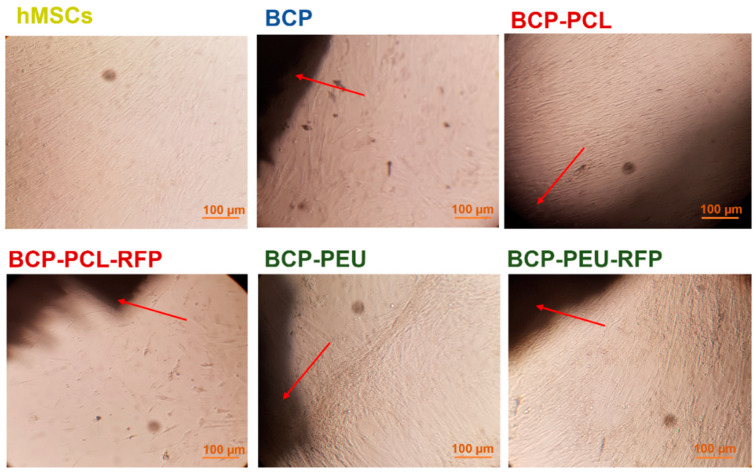
Images of hMSCs observed using an inverted microscope (scale bar, 100 μm), after 14 days of culture. Control group (cell culture plate), BCP scaffold, BCP-PCL scaffold, BCP-PCL-RFP scaffold, BCP-PEU scaffold, BCP-PEU-RFP scaffold.

The cytocompatibility of the obtained scaffolds in the hMSCs (a precursor of osteoblastic-lineage cells [31]) was monitored using MTS assay (cell viability and proliferation), and by morphological/adhesion studies using light and electron microscopes. According to the inverted microscope images (Figure 5), hMSCs seeded on the surface of the BCP-PCL, BCP-PEU, and BCP-PEU-RFP scaffolds showed similar or better cell growth than cells cultured on plates (untreated cells), presenting the fusiform morphology characteristic of this type of cells, after 14 days of culture. It can be observed that the hMSCs tended to grow towards the scaffolds (Figure 5). However, the cell density on BCP-PCL-RFP scaffolds was decreased and the morphology showed an unhealthy appearance (spheroidal shape) compared to the control group.

**Figure 6:**
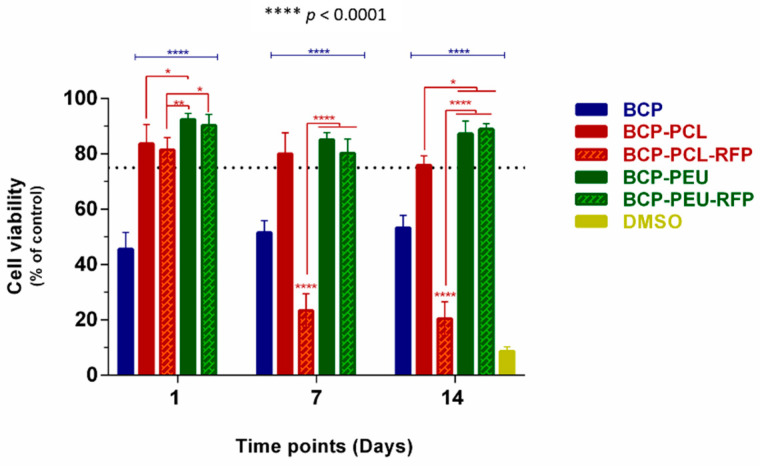
Cell viability of hMSCs onto BCP, BCP-PCL, BCP-PCL-RFP, BCP-PEU and BCP-PEU-RFP scaffolds. * *p* = 0.0432, ** *p* = 0.0008, **** *p* < 0.0001.

The results of cell viability (% relative to control—untreated cells) and proliferation (the optical density at 490 nm) of hMSCs seeded into unloaded and RFP-loaded scaffolds are displayed in Figure 6. No cytotoxicity to hMSCs was exerted by the coated scaffolds after 1 day of incubation when compared to the control group, irrespective of RFP loading, as can be deduced from the cell viability values of ~84% (BCP-PCL), ~92% (BCP-PEU), ~81% (BCP-PCL-RFP), and ~90% (BCP-PEU-RFP). However, after 7 days of culture, a significant decrease in cell viability to ~23% was observed for the BCP-PCL-RFP scaffolds. Similar results were obtained after 14 days. Compared to untreated cells, low cell viability (~50%) was registered at all time points for BCP scaffolds, as shown in Figure 6. Our results suggest that the BCP-PEU and BCP-PEU-RFP scaffolds exhibit a good level of cytocompatibility.

**Figure 7:**
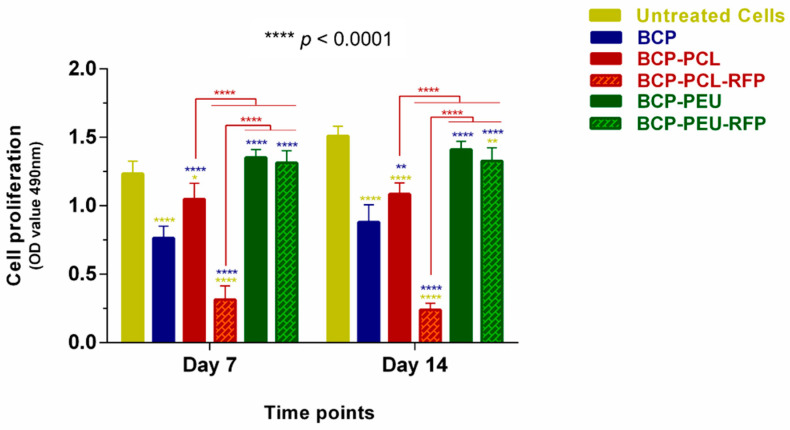
MTS assay for the proliferation of human mesenchymal stem cells (hMSCs) on the scaffolds cultured under different conditions on day 7 and 14. * *p* < 0.05; ** *p* = 0.0026, **** *p* < 0.0001.

Regarding proliferation studies, the MTS data after 7 and 14 days of culture showed that cell density increased with culture time in all scaffolds, except for the BCP-PCL-RFP one, which inhibited proliferation of hMSCs. As shown in Figure 7, there were no significant differences in the proliferation rate of hMSCs between the BCP-PEU/BCP-PEU-RFP scaffolds and control group (untreated cells) at days 7 and 14. Compared to the BCP-PCL scaffolds, the hMSCs proliferation levels were better in the BCP-PEU scaffolds and significant differences were observed (**** *p* < 0.0001). Similar results were found in the BCP-PEU-RFP scaffolds, suggesting that PEU coating promotes good cell adhesion and sufficient proliferation.

### 3.5. Cell Attachment

**Figure 8:**
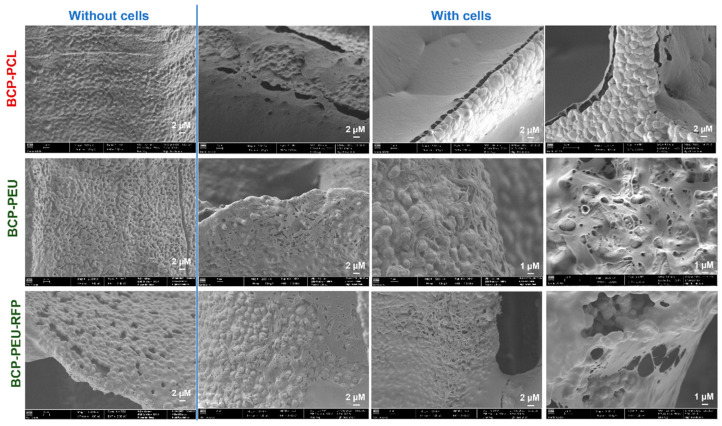
SEM micrographs of the surface of BCP-PCL, BCP-PEU and BCP-PEU-RFP scaffolds after polymer impregnation, without and with hMSCs after 14 days of culture.

Cell adhesion and morphology were examined by SEM. Figure 8 (without cells) shows SEM micrographs of the surface of the BCP-PCL, BCP-PEU, and BCP-PEU-RFP scaffolds after polymer impregnation, respectively, before the cell culture. In Figure 8 (with cells), SEM images of the surface of the BCP-PCL, BCP-PEU, and BCP-PEU-RFP scaffolds seeded with hMSCs cells, after 14 days of culture, are shown. SEM microscopic images (at low magnification) showed excellent adhesion and homogeneous distribution of hMSCs on the surface BCP-PCL, BCP-PEU, and BCP-PEU-RFP scaffolds, corroborating the MTS results. The surface of the scaffolds was almost completely covered by the cells and the extracellular matrix secreted by them. As can be seen in the high magnification images, a layer of cells and matrix on the surface of the scaffolds is observed. Regarding the morphology of the cells, it is difficult to withdraw conclusions.

## 4. Discussion

A complete conversion of CB into a BCP scaffold was obtained as reported in a previous work [17]. The unique CB architecture was retained after HT and sintering. Moreover, polymeric (PCL or PEU) coatings significantly improved the mechanical properties of the scaffolds without having a significant negative effect on porosity of the scaffold [17]. This means that the composite scaffolds skilfully combine the highly porous inorganic BCP with the polymeric coatings, improving the overall properties and expanding the application possibilities. Moreover, this synergistic effect can also be explored in the context of drug delivery. The main objective of this work was to develop a composite scaffold derived from CB that can act as a drug delivery system of an antibiotic, to avoid bacterial growth at the implantation site.

The discrepancy observed in the amounts of RFP incorporated into the scaffolds coated with the different polymers, 0.55 ± 0.04 wt% (PCL) and 0.45 ± 0.02 wt% (PEU), could be attributed to the higher viscosity of the PCL solution in comparison to that of PEU. This resulted in thicker PCL coatings [17], which are able to accommodate higher amounts of RFP. Due to the small amounts of RFP incorporated, it was not possible to detect differences between the coated samples with and without RFP using FTIR-ATR (Figure 2). It was also not possible to detect the presence of RFP in the samples using XRD and DSC (results in Supplementary Information, Figures S3 and S4). Nevertheless, its incorporation into the coated scaffolds can be easily deduced by the change of their colour from white to burnt orange (Figure 3). Moreover, the in vitro drug release studies (Figure 4) and the antimicrobial studies (Table 2) are other clear evidence of the presence of RFP in the coated samples.

The in vitro drug release studies (Figure 4) revealed that the applied polymeric coatings allowed for obtaining suitable RFP release profiles. All RFP loaded samples exhibited similar release profiles, characterized by an initial burst within the first 8 h, followed by a sustained release period. The relative amounts released during the burst period were 67.33 ± 1.48% and 47.23 ± 0.31% for PCL and PEU-coated samples, respectively. The incorporation of RFP into the scaffolds was aimed at preventing the risk of infection in the initial period after implantation. Therefore, the observed initial burst brings an important advantage to eliminate possible bacterial contamination during the medical procedure [32]. Moreover, it is important to highlight that RFP released during the burst period is still within the therapeutic limits, since a cytotoxic effect is observed only at a concentration of 100 µg.mL^−1^ [33]. This initial burst release may be due to different mechanisms, such as pore diffusion, surface desorption and the absence of a diffusion barrier regulating the diffusion process [34].

The Korsmeyer–Peppas model is suitable for describing the kinetics of drug release from thin films and cylindrical or spherical samples such as those used in this study. The drug release mechanisms can be divided into Fickian diffusion, non-Fickian transport and zero-order release when n is 0.45, 0.45–1 and 1, respectively [29]. For the different coating compositions used in the present work, the n value ranged from 0.46 to 0.62, which is characteristic of a non-Fickian diffusion. This diffusion behaviour indicates a release that is simultaneously controlled by diffusion and by dissolution [35].

The release profile is strongly dependent on the type of polymer coating. Indeed, a faster RFP release was observed from the scaffolds coated with PCL, compared to the scaffolds coated with PEU. The two different polymers, PCL and PEU, have carboxyl groups that allow the formation of hydrogen bonds with RFP. Moreover, these groups have affinity for calcium present in BCP scaffolds. PEUs, in addition to carboxyl groups, have amide groups that can also form hydrogen bonds with RFP, while having affinity for phosphorus from BCP scaffolds. Therefore, PEU coating is expected to produce stronger interactions with both BCP scaffolds and RFP compared to PCL. The lower affinity between PCL and BCP scaffolds results in weaker and non-continuous interfaces that provide a larger surface area for contact with the PBS solution and desorption of the drug. These factors explain and determine drug release behaviour and consistently account for its higher amount released from PCL coated samples within the first 8 h during burst period.

RFP is a broad-spectrum antibiotic for both Gram-positive and Gram-negative bacteria and was therefore chosen as the antibiotic in this work. The antibacterial activity data of the RFP-loaded scaffolds against Gram-negative *E. coli* and Gram-positive *S. aureus* reported in Table 2 confirm its useful action against both types of bacteria. However, the efficacy depends on the particular bacterial strain and also on the type of polymer coating. When incubated with *E. coli*, a more rapid decrease in the number of viable bacterial cells was recorded for the BCP-PCL-RFP samples. The BCP-PEU-RFP scaffold did not show antibacterial activity towards *E. coli*. This result can be associated with the higher amount of RFP released from the PCL-coated scaffold in comparison with the PEU-coated counterpart (Figure 4). Most probably the amount of RFP released from the BCP-PEU-RFP during the time of incubation is not sufficient to exert its antibacterial effect against *E. coli*. On the other hand, all the RFP-loaded scaffolds exhibited stronger antibacterial activity against *S. aureus*. In fact, the reduction of bacterial growth reached almost 100% after 24 h of incubation for all compositions. The higher sensitivity of *S. aureus* to RFP is associated with the better permeability of RFP through Gram-positive cell walls than through Gram-negative ones [36].

The in vitro studies performed with hMSCs support the suitability of the prepared scaffolds for bone regeneration during local antibiotic therapy. MTS metabolic assay showed that the unloaded and RFP-loaded PEU scaffolds, as well as the BCP-PCL scaffolds, could provide a favourable platform for the survival/growth of hMSCs. However, a low proliferation rate (~50%) was found in the presence of the uncoated BCP scaffolds, which proved to be much more fragile as compared to the coated ones. Similar results with BCP scaffolds were obtained by Kim and co-workers [37], with human osteoblast-like MG-63 cells, and of Hongmim and co-workers [38] with murine mesenchymal stem cells. This implies that the highly porous inorganic BCP scaffolds together with the polymer coatings (PCL and PEU) play a synergistic role, by exhibiting less or no cytotoxicity and providing the suitable conditions for cell adhesion and proliferation. Compared to the BCP-PEU scaffolds, the PCL coated BCP scaffolds showed low proliferation and viability values over the experimental period (7 and 14 days). These findings could be due to a reduction in the porosity of the BCP-PCL scaffold, which consequently leads to a reduction in the total surface area available for cell adhesion [17]. Similar biological observations were reported by Milovac and co-workers [39] and Siddiqi and co-workers [40] for the PCL coated BCP scaffolds. Moreover, the BCP-PCL-RFP scaffolds were shown to negatively affect the viability and proliferation of hMSCs (i.e., higher cell toxicity and lower cell proliferation rate), resulting cell damage. This result, however, can be related with the higher amount of RFP released, after 1 day, from the PCL-coated scaffold (≈80%) in comparison to the amount of drug released from PEU-coated scaffold (≈50%). The higher amount of RFP released can induce toxicity on hMSCs, affecting their viability and proliferation. In contrast, cells proliferating on the surface of BCP-PEU-RFP scaffold were not negatively affected. The results revealed that BCP-PEU-RFP scaffolds have excellent properties for the intended applications, namely: cytocompatibilitys, and an optimal composition to support cell proliferation.

## 5. Conclusions

In this study, a drug delivery system using composite scaffolds, from BCP, coated with biopolymers and loaded with a pharmaceutical drug (RFP), was successfully developed. The polymeric (PCL or PEU) coatings bring synergistic benefits to the mechanical properties of the scaffolds [17], opening also the possibility of the scaffold to be used as a delivery system. The drug release kinetic mechanism was shown to be a non-Fickian diffusion. The RFP release from the BCP-coated scaffolds was similar for both coatings (PCL and PEU), being characterized by a burst release in the first 8 h, followed by a sustained release. A higher initial release of RFP from the PCL-coated samples could be attributed to the weaker interaction between the polymer and the drug and also between the polymer and the inorganic BCP scaffold. The antibacterial activity of the different scaffolds against *E. coli* and *S. aureus* showed that the samples without the antibiotic had no antimicrobial activity. Both RFP-loaded scaffolds were effective in reducing bacterial growth, particularly in the case of *S. aureus*. Regarding *E. coli*, only BCP-PCL-RFP was shown to have antibacterial activity. The in vitro cytotoxicity studies revealed that hMSCs adhered to the surface of BCP-PEU-RFP scaffolds, proliferated, and remained viable after 7 and 14 days of culture. Overall, the results show that the BCP-PEU-RFP scaffolds are the most promising for the application.

## Figures and Tables

**Table 1:** Exponent (*n*), constant (*k*) and correlation coefficient (*R*^2^) obtained from the Korsmeyer–Peppas model for the release of RFP from the BCP scaffolds coated with PCL and PEU.

Scaffold	*n*	*k*	*R* ^2^
BCP-PCL-RFP	0.6215	0.3058	0.9807
BCP-PEU-RFP	0.4640	0.1841	0.9890

**Table 2:** Antibacterial activity of the different scaffolds. The antibacterial activity was determined by following the number of viable cells as CFU ml^−1^.

***E. coli***				
**Scaffold**	**24 h**	**48 h**	**72 h**	**Attached Cells**
BCP-PCL	>3 × 10^3^			
BCP-PCL-RFP	4.25 ± 0.25	0.00 ± 0.00	0.00 ± 0.00	6.00 ± 6.00
BCP-PEU	>3 × 10^3^			
BCP-PEU-RFP	>3 × 10^3^	>3 × 10^3^	>3 × 10^3^	>3 × 10^3^
***S. aureus***				
**Scaffold**	**24 h**	**48 h**	**72 h**	**Attached Cells**
BCP-PCL	>3 × 10^3^			
BCP-PCL-RFP	0.00 ± 0.00	0.17 ± 0.24	0.00 ± 0.00	0.00 ± 0.00
BCP-PEU	>3 × 10^3^			
BCP-PEU-RFP	0.17 ± 0.24	0.00 ± 0.00	0.33 ± 0.47	10.00 ± 10.50

## Data Availability

The data presented in this study is available in the article.

## Supplementary Materials

The following are available online at https://www.mdpi.com/article/10.3390/polym13244378/s1, Figure S1. Calibration curve of RFP diluted in DMSO, Figure S2. Calibration curve of RFP diluted in PBS, Figure S3. XRD of uncoated BCP scaffolds, coated with different polymers (BCP-PCL, and BCP-PEU) and loaded with RFP (BCP-PCL-RFP and BCP-PEU-RFP), Figure S4. DCS of uncoated BCP scaffolds, coated with different polymers (BCP-PCL and BCP-PEU) and loaded with RFP (BCP-PCL-RFP and BCP-PEU-RFP).
