# Supplementary material for: Highly Porous Composite Scaffolds Endowed with Antibacterial Activity for Multifunctional Grafts in Bone Repair
Source: Polymers (Basel). 2021 Dec 14;13(24):4378. doi: 10.3390/polym13244378 (PMC8705097; doi:10.3390/polym13244378)
Supplement: Supplementary file 1 [file polymers-13-04378-s001.zip › polymers-1467629-supplementary.pdf]

Supporting Information for:

Highly porous composite scaffolds endowed with antibacterial activity for multifunctional grafts in bone repair

Ana S. Neto[1], Patrícia Pereira[2,3], Ana C. Fonseca[2], Carla Dias[4], Mariana Almeida[4], Inês Barros[5,6,7], Catarina O. Miranda[5,6,7], Luís P. Almeida[5,6,7,8,9], Paula Morais[4], Jorge F. J. Coelho[2,*], José M. F. Ferreira[1*]

[1] Department of Materials and Ceramic Engineering / CICECO – Aveiro Institute of Materials, University of Aveiro, 3810-193 Aveiro, Portugal;
[2] CEMMPRE, Department of Chemical Engineering, University of Coimbra, Rua Sílvio Lima-Pólo II, 3030-790 Coimbra, Portugal;
[3] IPN, Instituto Pedro Nunes, Associação para a Inovação e Desenvolvimento em Ciência Tecnologia, Rua Pedro Nunes, 3030-199 Coimbra, Portugal;
[4] CEMMPRE, Department of Life Sciences, University of Coimbra, 3001-401 Coimbra, Portugal;
[5] CNC - Center for Neuroscience and Cell Biology, University of Coimbra, Portugal;
[6] CIBB - Center for Innovative Biomedicine and Biotechnology, University of Coimbra, 3004-504 Coimbra, Portugal;
[7] IIIUC - Institute for Interdisciplinary Research, University of Coimbra, 3030-789 Coimbra, Portugal;
[8] Faculty of Pharmacy, University of Coimbra, 3000-548 Coimbra, Portugal;
[9] Viravector - Viral Vector for Gene Transfer Core Facility, University of Coimbra, 3004-504 Coimbra, Portugal
* Correspondence: A. C. Fonseca (anafs@eq.uc.pt), J.M. Ferreira (jmf@ua.pt)

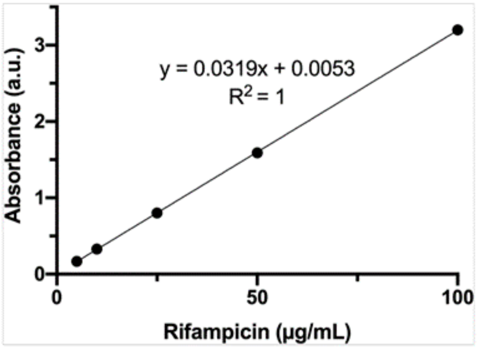

Figure S1: Calibration curve of RFP diluted in DMSO.

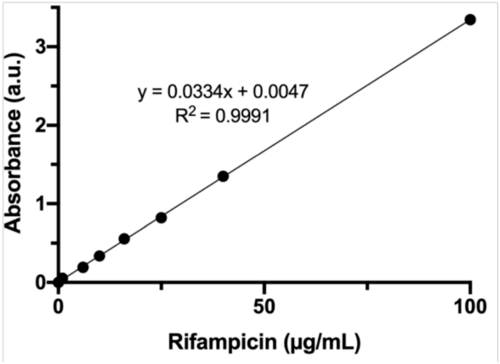

Figure S2: Calibration curve of RFP diluted in PBS.

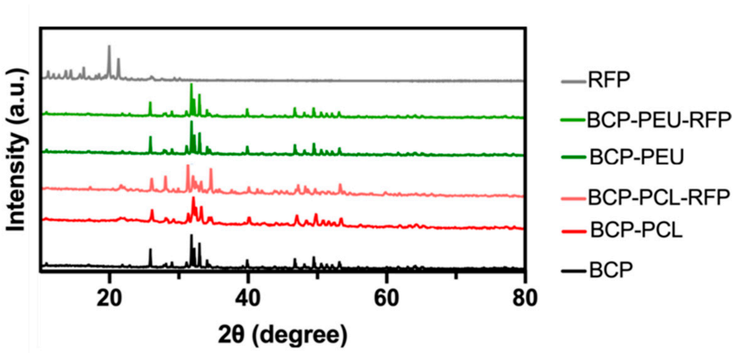

Figure S3: XRD of uncoated BCP scaffolds, coated with different polymers (BCP-PCL, and BCP-PEU) and loaded with RFP (BCP-PCL-RFP and BCP-PEU-RFP).

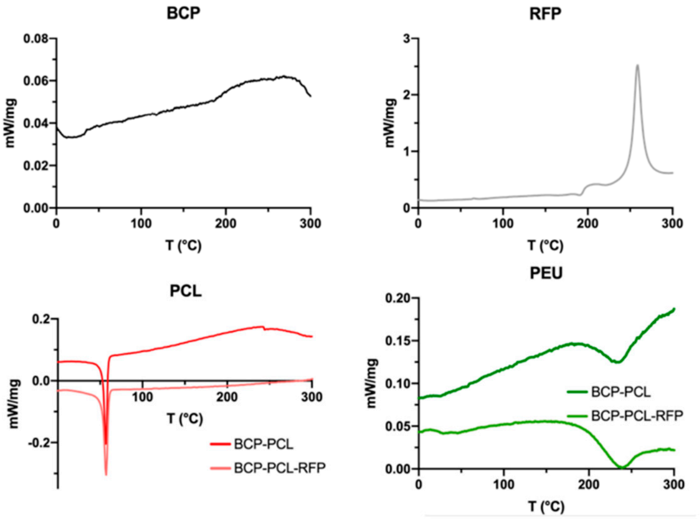

Figure S4: DCS of uncoated BCP scaffolds, coated with different polymers (BCP-PCL and BCP-PEU) and loaded with RFP (BCP-PCL-RFP and BCP-PEU-RFP).
